# Intramuscular myxoma of the hyoglossus muscle: A case report and literature review

**DOI:** 10.3892/ol.2014.1955

**Published:** 2014-03-07

**Authors:** GUIQI LI, WEN JIANG, WEI LI, JUNCHUAN LI

**Affiliations:** 1 Department of Stomatology, The First Affiliated Hospital of Yangtze University, Jingzhou, Hubei 434000, P.R. China; 2 Department of Pathology, The First Affiliated Hospital of Yangtze University, Jingzhou, Hubei 434000, P.R. China

**Keywords:** intramuscular myxoma, hyoglossus muscle, benign tumor

## Abstract

Intramuscular myxoma (IM) is a benign intramuscular neoplasm composed of fibroblasts and abundant myxoid stroma. IMs most commonly affect larger skeletal muscles, while those affecting the oral and maxillofacial regions are rare, with a small number of documented cases in the available literature. The aim of the present study was to describe a highly rare case of an IM within the hyoglossus muscle of the tongue in a 74-year-old male. The patient presented with a painless mass in the submental space that had been growing slowly for more than five years. A computed tomography scan revealed a hypodense lesion located in the root of the tongue. The mass was easily excised with thin margins, including only a small amount of the adjacent muscle tissue. The pathological diagnosis of the mass was an IM. The patient made an excellent recovery following the surgery and the follow-up three years later revealed no local recurrence. IMs of the hyoglossus muscle are highly rare, however must be considered in the differential diagnosis of swellings in the root of the tongue region.

## Introduction

Myxoma is a benign tumor of mesenchymal origin composed of undifferentiated stellate cells in a loose mucoid stroma with delicate reticulin fibers. The diagnostic criteria of the myxomas were initially proposed by Stout (1). These tumors develop in a variety of locations, including the heart, subcutaneous and aponeurotic tissues, bones, genitourinary tract, skin, retroperitoneum, intestine, pharynx, joints and skeletal muscles. Myxomas that arise from skeletal muscles are known as intramuscular myxomas (IMs), which were described as a distinct subtype of myxomas in 1965 by Enzinger (2), constituting only 17% of all soft tissue myxoma cases in his study. IMs have an incidence of ~1/1,000,000/year, most commonly occur in females, with a ratio to male patients of 14:3 (3) and have an adult predominance, with only two cases reported in infants (4). Myxomas are uncommon in the oral and maxillofacial region, and develop almost exclusively in the jaw, where they are considered to be of odontogenic origin. By contrast, soft tissue myxomas rarely occur in the oral and maxillofacial region, with IMs that do present in the oral and maxillofacial region being extremely rare. Following a review of the literature, it was revealed that only nine cases of IM in these regions have been documented (Table I). In the present report, the case of a 74-year-old male with an IM in the hyoglossus muscle of the tongue is described, with a brief review of the literature concerning this condition. Patient provided written informed consent.

## Case report

A 74-year-old male patient presented to the First Affiliated Hospital of Yangtze University (Jingzhou, China) with a painless mass in the anterior region of the upper neck that had been growing slowly for more than five years; the patient noted that the expansive mass had exhibited a progressive volume increase within the last six months. The patient also complained that swallowing food and pronunciation had been affected by the mass. The patient had no history of trauma, fever or weight loss. Clinical examination revealed a firm and tender mass (diameter, ~8 cm) in the root of the tongue.

**Figure 1:**
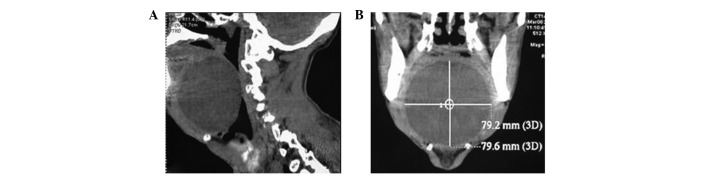
Cervical CT scan reveals a separated, well-defined and hypodense mass in the root region of the tongue. (A) The mass located within the hyoglossus muscle of the tongue and upon the hyoid bone. (B) Cervical CT scan reveals the diameter of the mass. CT, computed tomography.

Computed tomography (CT) of the neck revealed a hypodense lesion located in the anterior cervical region of the neck, on the hyoid bone within the hyoglossus muscle. The CT value of the tumor was ~20.3 HU (Hounsfield unit). The oropharyngeal cavity became narrow as a result of tumor pressure (Fig. 1A) and the diameter of the mass was ~80 mm (Fig. 1B). The imaging diagnosis characterized the mass as a cystic space-occupying lesion or lipoma.

Surgical resection was accomplished through a horizontal incision on the hyoid bone over the tumor site, under general anesthetic. The tumor was easily separated from the normal muscle margins of the root of the tongue and the mucous membrane of the root of the tongue, which was adhered to the tumor was resected.

**Figure 2:**
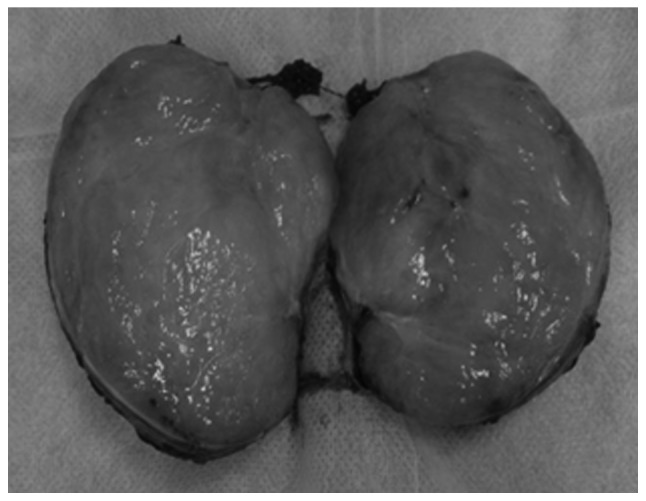
Postoperative macroscopic observations of the tumor; the cut surface of the specimen was ovoid, yellow in color and gelatinous.

**Figure 3:**
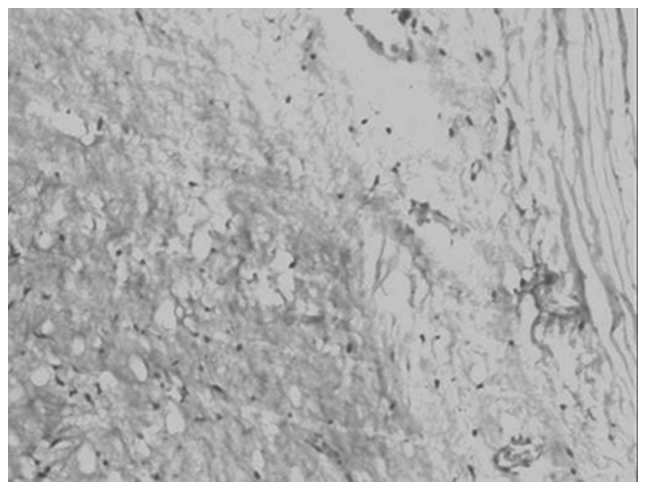
Histopathological images of the intramuscular myxoma. Photomicrography demonstrates the capsule of the tumor and reveals a myxoid stroma sparsely populated by small spindle-shaped cells, stellate cells and fibers (hematoxylin and eosin staining; magnification, ×100).

The tumor was encapsulated intact and macroscopically, it exhibited a gray-white appearance and the cut surface of the specimen was ovoid, yellow and gelatinous (Fig. 2). Histopathological analysis revealed a hypocellular neoplasm of low vascularity, composed of small spindle-shaped cells, stellate cells and fibers in abundant myxoid stroma. Mitotic activity, necrosis and nuclear atipias were absent. The fibrous pseudo-encapsulation of the tumor was identified and there was no infiltration of the fascicles of the adjacent skeletal muscle. The neoplastic cells were negative for S-100 protein expression. The pathological diagnosis of the mass was an IM (Fig. 3). The patient experienced an excellent recovery following surgery and was able to swallow food and speak without pronunciation issues. The follow-up at three years demonstrated no evidence of local recurrence.

## Discussion

In 1871, Virchow (14) used the term myxoma to describe a lesion that resembled the mucinous substance of the umbilical cord. The diagnostic criteria of myxomas were first proposed by Stout (1) and IMs were described as a distinct subtype of myxomas in 1965, by Enzinger (2). IM is a slow-growing tumor and usually presents as a painless mass that may exhibit symptoms due to the compression of surrounding structures (2). The most frequent sites of IMs are the muscles of the thigh, buttock, shoulder, lower leg, arm and trunk (2,5). IM in the oral and maxillofacial location is highly rare. Head and neck intramuscular myxomas usually occur in patients between the ages of 40 and 60 years (15). In the present case, the patient had experienced no pain as a result of the mass for five years, however, in the six months prior to diagnosis, the mass began to interefere with the patient’s swallowing and pronunciation as the oropharynx had been compress by the large mass.

The clinical diagnosis of IM is problematic prior to microscopic examination due to the oral and maxillofacial location, as IM exhibits non-specific clinical manifestations. Therefore, the clinical differential diagnosis includes benign tumors of mesenchymal origin, for example, benign muscle neoplasms, such as rhabdomyoma and leiomyoma. In the present case, the patient’s tumor was located in the root of tongue, therefore various diagnoses could have been considered, including a more common benign tumor or cyst of the tongue, such as a dermoid or epidermoid cyst, teratoma or neurilemmoma. The mass may also have been a rare benign tumor, such as a tumor arising from the lingual ectopic thyroid or lipoma, or it may have been a benign salivary gland tumor (pleomorphic and monomorphic adenoma) arising from the small salivary glands of the root of the tongue.

The majority of tumors are solitary, however, a small proportion are multiple and are associated with fibrous dysplasia. The combination of multiple tumors, or more rarely solitary IMs, with skeletal fibrous dysplasia in now termed Mazabraud’s syndrome (16); when an IM is suspected, the patient must also be examined for fibrous dysplasia.

IMs lack specific radiographic features and CT scans typically reveal a cystic-like mass, with a CT value that is greater than water, but less than the surrounding normal muscle (17). These radiological features may be presented in other lesions, such as cystic hygroma, lipoma and cystic teratoma amongst others. Therefore, it is difficult to preoperatively diagnose IM using CT scans, and as a result these tumors are frequently misdiagnosed as cystic hygroma or lipoma. However, CT scans of these masses is a necessity, as they provide information regarding the structure of the lesion, including the tumor size, boundaries and the associations between the tumor and the surrounding tissues. In the present case, the CT scan revealed that the mass had sharp borders, with no infiltration of the adjacent muscle and the tumor was identified as benign. Imaging diagnosis identified the mass as a cystic space-occupying lesion of the root of the tongue.

Due to a lack of specific symptoms and common laboratory tests for identifying IMs, the diagnosis of IMs is difficult. It is very rare for these tumors to be correctly diagnosed prior to biopsy and histological examination. It has been reported that carbohydrate antigen (CA) 19-9, a tumor marker, may be correlated with IM. In a previous study, the serum level of CA 19-9 increased preoperatively and returned to a normal level six months following surgery (18), however, the levels also increased in a variety of other malignant and benign conditions. The origin and the nature of the tumor can be established via fine needle aspiration, however, while the diagnosis of an IM is possible using this method (8,19), the final diagnosis is always based on the histopathological examination.

Macroscopic analysis of IMs has demonstrated that the majority of these tumors are ovoid or globular, and covered by bundles of skeletal muscle, with a yellow/gray and gelatinous cut surface. Microscopic visualization using hematoxylin and eosin staining, shows that IMs are hypocellular, hypovascular, intensely mucoid and basophilic, and are composed of stellate and spindle-shaped cells in a myxoid stroma; certain IMs exhibit focal areas of hypercellularity and hypervascularity (20,21). Hypercellular zones appear in 76% of IMs and may occupy 10–80% of the tumor (20) and in these hypercellular zones, an absence of mitoses, nuclear atypia and necrosis indicates IM (2,20). In one immunohistochemical analysis, the neoplastic cells were positive to vimentin and cluster of differentiation 34, however, were negative for S-100 protein and smooth muscle actin (3,22).

IMs present as benign masses and there have been no reports of cases involving metastasis or other malignant changes. As a result, the treatment for IM is surgical excision, including enucleation, simple excision and wide local excision. Previous studies demonstrated several cases of recurrence following incomplete excision or simple enucleation due to the adjacent muscle tissue being infiltrated or an incomplete capsule (20,23,24). However, there were no recurrences of the solitary IMs following enucleation, simple excision or wide local excision (11,23,25) and simple excision (with a margin of only a few muscle fibers) is highly recommended (12,13). In the present case, total removal of the tumor was performed by simple excision, and the mucous membrane of the root of the tongue, which was adherent to the tumor capsule, was resected. There were no signs of recurrence three years following surgery, therefore, simple excision is considered to be a feasible method.

In conclusion, the occurrence of IM in the hyoglossus muscle is highly rare. To the best of our knowledge, this is the first study of this type of tumor in this region. An accurate diagnosis prior to surgery is difficult due to a lack of characteristic clinical history and radiographic findings; therefore, a CT scan or MRI is required for treatment planning. Furthermore, IM must be considered in the differential diagnosis of swellings in the root of the tongue and simple excision was identified as a feasible method for the treatment of solitary IM.

## Figures and Tables

**Table I:** Cases of intramuscular myxoma in the oral and maxillofacial region, as reviewed in the literature.

Case	First author, year (Ref.)	Location	Age/Gender
1	Rosin RD, 1973 (5)	Geniohyoid muscle	44/M
2	Bedrosian SA*,* 1984 (6)	Masseter muscle	43/F
3	Nishijima W, 1985 (7)	Digastric muscles	16/F
4	Mockli GC, 1993 (8)	Tongue	
5	Serrat A, 1998 (9)	Temporalis muscle	
6	van Roggen, 2001 (10)	Right cheek	56/M
7	Robin C, 2004 (11)	Temporalis muscle	43/F
8	Papadogeorgakis N, 2009 (12)	Masseter muscle	74/M
9	Patsiaoura K, 2009 (13)	Mimetic muscles of the nasal vestibule	52/M

M, male; F, female.
